# Mathematical Biology in *Biology Direct*

**DOI:** 10.1186/1745-6150-3-1

**Published:** 2008-01-16

**Authors:** Andrei Yakovlev

**Affiliations:** 1 Department of Biostatistics and Computational Biology, University of Rochester, Rochester, New York 14642, USA

## Editorial

The last decade has seen a dramatic change in the status of mathematical methods in biology. The usefulness of mathematics in providing a deeper insight into the organization of fundamental biological processes is now widely recognized. Many areas of modern biology are becoming increasingly quantitative. In my opinion, however, this process is far from optimal. There is still a tangible proportion of *ad hoc *methods lacking theoretical underpinnings and/or rigorous testing. Biological systems are extraordinarily complex, calling for methods that are commensurate with this complexity. Modern mathematics offers a much richer arsenal of tools and ideas than those that are frequently employed to describe the enormous diversity of biological phenomena. This does not imply that all methods of data analysis should necessarily be sophisticated but they must meet a much higher standard of scientific rigor than is currently the case. The new Mathematical Biology section of *Biology Direct *is intended to help set such a standard.

We welcome research and review papers dealing with all potentially interesting biological applications. Critical comments both of a general nature and specifically related to previously published papers represent another important category of contributions. Mathematically and computationally intensive approaches may lead to significant biological discoveries. In some cases, these discoveries are conducive to very concise presentation, and the most appropriate venue for this type of publication is provided by "Discovery Notes".

On a personal note, I am truly enthusiastic about the new section not only because of great expectations for future contributions but also because of the alternative publication concept set forth by *Biology Direct*. Many flaws in the traditional system of anonymous peer review are well-known. Why is it possible that erroneous papers make their way to publication in a peer-reviewed journal despite its "high-profile" status? Why are our peers always amicable and reasonable when discussing scientific issues at conferences and seminars but, often, much less so when reviewing papers submitted by their competitors and critics? Why is it sometimes apparent that the reviewer does not even care to read the paper? Or why is it sometimes clear that the reviewer is unable to grasp the essentials of the problem under consideration, and yet, rejection is recommended? The most natural explanation is that the anonymous peer review system is too amenable to abuse of power. *Biology Direct *is one of the very few journals, and perhaps, the only interdisciplinary journal in today's biology that strives to find a cure for this problem. (It is my understanding that the policy adopted by *PLoS ONE *that has proclaimed similar goals is much less impartial as it places the main emphasis on pre-review decision-making by members of the editorial board who may or may not be experts in the field). However imperfect the proposed solution might seem, it drives the reviewers and authors alike to act in a more responsible way. Much like politics, science needs openness and transparency. Personal agendas and special interests must be set aside when both the authors and the reviewers are trying to find the truth – this is the main thrust of the new concept.

There is no doubt in my mind that the new concept of an open review process will eventually succeed, and I am determined to help its growth as best I can. *Biology Direct *needs constant support from those who believe in real scientific progress and whose interests are not limited to career goals, social relations, and research funding. It is not a high rejection rate that makes a good journal but the ability to attract scientifically rigorous, innovative and clever works. I intend to submit my best future papers not to the traditional high-impact journals but to *Biology Direct *in an effort to make a difference in the inherently flawed publishing system. I hope that many true scientists support this effort.

